# An Audit to Assess the Quality of Ward Referrals Sent to City Hospital Liaison Psychiatry Team From Inpatients Wards D15, D17and D27, Between July 2021 to September 2021

**DOI:** 10.1192/bjo.2022.483

**Published:** 2022-06-20

**Authors:** Kesegofetse Setlhare, Hannah Woodman, Amandeep Pahal, James Hickmott

**Affiliations:** 1 Black Country Healthcare NHS Foundation Trust, Wolverhampton, United Kingdom; 2 Sandwell and West Birmingham Hospitals NHS Trust, Birmingham, United Kingdom

## Abstract

**Aims:**

Liaison psychiatry provides psychiatric care to medical patients. Patients include those attending emergency departments, general hospital inpatients and outpatients. Liaison teams work hand in hand with several general hospital teams to offer advice, review and manage these patients. Over the last few months, the Liaison service in City Hospital have been receiving many inappropriate referrals. Inappropriate referrals are defined as patients who are referred to services, with one of the following reasons:

1. Insufficient presenting complaint
2. No documented Past psychiatric history
3. Insufficient Mental state Examination (MSE)
4. No risk assessment
5. No documented Drug/alcohol history
6. Patients having not consented to referral.
7. If one or more of the above criteria is not met

Our aim was to evaluate the appropriateness of the referrals received from D15, D17, D27 inpatients wards in City Hospital over a 3-month period from July to September 2021. These wards were chosen as they commonly refer patients to liaison services.

**Methods:**

We collated data retrospectively on the nature of all referrals from D15, D17 and D27 ward over a 3-month period. The patient referral portal was used, and referral content of each patient was analysed. An audit tool was devised to assess whether the referrals followed the liaison referral pathway and guidelines set by NHS England for referral structure to liaison services.

**Results:**

18 patients were referred to the Liaison psychiatry from the three wards over the three-month period. We observed 77.8% (n = 14) of the referrals having insufficient information for the presenting complaints, whilst 22.2% (n = 4) of them did not state past psychiatric history. Approximately 94.4%(n = 17) did not state sufficient details of MSE. In 83.3% (n = 15) of referrals appropriate detailed risk assessment was not done, 27.8% (n = 5) of them did not have alcohol/ drug use stated and 22.2% (n = 4) of patients referred did not consent to the referral being made.

**Conclusion:**

The results demonstrated that ward referrals lack quality and contain inadequate information to allow for safe screening of patients and for the implementation of appropriate actions by the liaison team. A possible reason for inappropriate referrals may be due an existing knowledge gap and lack of confidence taking detailed psychiatric histories, assessing risk, and performing MSE in non-psychiatric trainees making referrals to liaison services.

